# The power of connected clinical teams: from loneliness to belonging

**DOI:** 10.1186/s13010-023-00143-7

**Published:** 2023-11-09

**Authors:** Jacqueline Hoare

**Affiliations:** Department of Psychiatry and Mental Health, University of Cape Town, Anzio Road Observatory, Cape Town, 7925 South Africa

**Keywords:** Medical culture, Mental health, Psychological safety, Vulnerability, COVID-19

## Abstract

**Background:**

We need to preserve the lessons of the COVID-19 pandemic in caring for the mental health of clinicians, of shared experiences, interdependence, team cohesion and vulnerability, among others. We need reform in the way that clinicians are cared for, and a resistance to the idea of a post-pandemic ‘return to normal’.

**Main text:**

To build connected and optimally functioning clinical teams, we need to create an inclusive culture in which difficult conversations and caring are the expectation. If we are to be better at solving problems and better at serving our patients, we should be vigilant about creating a psychologically safe medical culture in which colleagues feel safe, seen, heard, and respected.

**Conclusion:**

Going forward, each of us, regardless of seniority, needs to take responsibility for this culture change. We need to create and participate in weekly collegial peer support sessions that feel nurturing and safe, that allow us to reveal parts of ourselves, to be vulnerable with each other in a way that reduces loneliness, and encourages and maintains social connections and a sense of belonging within clinical teams, improves clinician well-being and reduces the risk of burnout.

*“Care is a practice of informed responsive actions on behalf of the one cared for and authentically aimed toward their growth and flourishing.”* *Care Ethics in the Age of Precarity; Maurice Hamington and Michael Flower.*

## Background

The prevalence of depression, anxiety, insomnia, and PTSD in clinicians from Sub-Saharan Africa during the COVID-19 pandemic has been high [1]. A systematic review of healthcare worker distress following infectious disease outbreaks has identified that their distress may continue for several years [2]. Work-related psychological distress in health workers may be associated with long-term negative adverse effects such as decreased quality of patient care, conflict with colleagues, cognitive impairment, substance use, suicide, poorer physical health, and leaving the healthcare profession. In the run-up to the COVID-19 pandemic, there was recognition that clinicians were overstretched, under-resourced, and chronically distressed [3]. The COVID-19 pandemic has highlighted the need to reassess and reconstruct the culture of our healthcare and academic institutions if we are to ensure the long-term well-being of clinicians and researchers [4]. Unlike sports teams, there is no reserve team of healthcare workers waiting on the side-lines to substitute those on duty so that the current shift can rest [4]. I use my experience as a Consultation Liaison Psychiatrist at a large teaching public hospital in Cape Town, South Africa to reflect on key lessons for supporting clinicians in challenging and limited resource contexts. Although this essay narrates my participation in supporting clinicians in a Sub-Saharan African university public hospital, I maintain that lessons learned here have international merit and are relevant to most healthcare institutions.

## Main text

### Disconnection, lack of community, loneliness, and burnout

The traditional medical workplace where a rigid hierarchy model of leadership has dominated is now outdated. Power concentrated in the hands of few can be wielded indiscriminately against early career researchers and clinicians, creating an environment that compromises psychological safety and destroys the open and collaborative culture that produces good science and clinical care. The culture of medicine frequently reinforces that self-care is selfish, and that physical and emotional exhaustion are to be expected [5]. In addition, the belief that vulnerability is a sign of weakness in medical culture is reinforced regularly. Despite the importance of psychological safety in healthcare teams, it is often lacking [6]. When teams are psychologically safe, they have a shared belief that they can take interpersonal risks such as speaking up, asking questions, and sharing [6]. A lack of psychological safety destroys the open and collaborative culture that produces good patient care and drives talented individuals out of medicine [7].

Clinicians tend to feel isolated and lonely in their vulnerability and suffering [8]. This belief is reinforced by a culture of silence which convinces clinicians that their colleagues are successfully managing these stresses. Studies have also shown that many clinicians struggle to tell their colleagues or employers about their mental health problems [9]. The most commonly cited reasons are perceived stigma, and anticipated damage to future career [10]. Suicidal ideation in clinicians can present strong fears of stigmatisation [11]. Such concerns may be underpinned by feelings of shame and professional failure, and associated worries about fitness to practise [12]. Social isolation and loneliness (the subjective and distressing feelings stemming from a subjective feeling of disconnection) co-occur frequently, but not necessarily [13]. Both independently contribute to the risks of poorer health, including substantial increases in the risk of many medical issues, and strong evidence demonstrating increased risks of cardiovascular disease and death from any cause [13]. Many studies have shown that those experiencing loneliness have an increased risk of anxiety and depression, dementia, infectious diseases, poor functioning, and death from overdose or suicide [13]. Problems arise within clinical teams at healthcare facilities when feelings of loneliness or being disconnected becomes chronic. A high rate of loneliness in medical settings has been correlated with poor work organization, less managerial support, worse atmosphere in the team, and more irresponsible attitudes of colleagues [14]. Loneliness in clinicians can be used to predict occupational burnout [15, 16].

Historically, clinician well-being efforts have overemphasised resilience programmes, thereby placing the burden of managing emotional distress on individual clinicians themselves [5]. Programmes relying on individual clinician referrals often fail because they require clinicians to recognize and name that they are struggling and need support [5]. Stigma, shame, and isolation are substantial barriers to the success of these individual focused well-being efforts [5].

### The COVID-19 pandemic facilitated dependence on teams and more shared vulnerability

I think we are at risk of misunderstanding the challenge if we think that the awareness and need of support, connection, and belonging should return to how they were before COVID-19. I would argue that the goal is not only recovery of the ravages of the waves, but also learning lessons for new ways of working together. A positive working culture is diverse, collaborative, transparent where contributions feel supported, individuals seen, and innovation is given space to flourish [7]. I have learned that teamwork and ‘walking alongside each other’ was welcomed by clinicians. We took time to listen to and care for each other [8]. I counsel taking steps to avoid losing these important aspects of the culture in which we worked during COVID-19. Attention should be sustained regarding maintaining the coherence of clinical teams in the face of ongoing pressures [4]. Mental health professionals are already convinced of the therapeutic value of being able to tell one’s story, especially to those who have had similar experiences: this type of social support in healthcare settings can help to reduce feelings of isolation, which compound loneliness and trauma [8]. I often reflect on the nature, structure, and function of human connections in the hospital in which I work, a function I have found to be indispensable to my own mental health. Being connected to others around us is considered a fundamental human need, crucial to health and wellness [17]. Yet loneliness and a lack of belonging in the hospital setting is such a hard thing for clinicians to admit to. Admitting to loneliness feels uncomfortable, and the discomfort makes us want to move on to a different topic. However, willingness to ask uncomfortable questions, like those enquiring about loneliness, can lead to great conversations [17]. This is how we can build relationships between clinicians in our hospitals, how we reach out to others who are feeling lonely, and how we create a community of inclusion. The key lesson from our time spent working on the COVID-19 frontline was ‘the power of a connected clinical team’. The sense of being connected to each other and the experience around us, the sense of belonging, the sense of safety created by the teams is something that we should hold onto post-pandemic.

### Weekly peer support groups on protected, paid time, structured to help facilitate vulnerability, connection, and belonging

Changing the public healthcare system in limited resource settings, in which primarily uninsured patients are cared for, is complicated. It is something outside the scope of the clinicians who simply show up for work every day. We can’t control the number of patients who arrive at our doors daily or change the economic and social settings of our hospitals. However, part of the solution could be setting up a system where clinicians can contribute to a change in medical culture by changing how they relate to each other, talk to each other about their experiences, work-related stress and teams’ psychological safety can be strengthened [18]. Previous research has established the benefits of improving psychological safety in healthcare teams, and it is time to build interventions that do so [6]. All clinicians, regardless of seniority, should be calling for peer support groups to be integrated into their working week. It is clear that, when necessary, it was possible to liberate clinicians to enable them to attend group sessions, despite our limited resource setting, and this should continue. During and after the COVID-19 pandemic, I have been running weekly Balint-style support groups (60–90 min) in a variety of departments in my hospital, including the departments of medicine, critical care, and anaesthetics. The size of the groups ranged from 5 to 20 participants in each group per week. The size of the groups varied due to issues such as shift rotas, annual and sick leave, as well as clinicians required to attend emergencies. Advantages of the Balint group in medical institutions can be the improvement of the doctor-patient relationship, the decrease of burnout, increased empathy, management of stress and anxiety, and positive attitudes and perception about group interactions and colleagues [19]. The sessions facilitated difficult conversations during which clinicians could openly discuss their feelings of frustration with, for example, needing to navigate how to engage with patients who are ambivalent about following scientific evidence for COVID-19 prevention [20]. The conversation topics were determined by the group members, while my role was to facilitate the discussions only. I also believe that what was most helpful is that I did not try to fix everything or tell colleagues what to do. In societies characterized by deep inequality and violence, such as ours in South Africa, the environment in which clinicians work is distressing in itself. This compounds the experience of moral distress in healthcare as resource shortages in the system combined with patients’ very difficult personal circumstances leads to frequent exposure to distressing ethical situations [21]. It is also important to facilitate a space to think through complex, ethical, and moral dilemmas and issues at risk of causing moral injury – a stressor that clinicians are particularly vulnerable to.

Key to my intervention, furthermore, was the fact that I took the decision to work directly with groups of doctors in the COVID-19 medical wards. The groups could happen, and could be useful, precisely because I become part of the COVID-19 team, which meant that I was dealing with the same trauma and anxieties myself, while bearing witness to the same suffering and deaths that they were [8]. This ‘shared experience’ made it possible to shift the model of helping my colleagues from a traditional group therapy model to being a collegial support space for sharing of trauma. It became possible to safely express emotions in the groups because everyone had been through the same trauma. Providing protected time to reflect on the personhood of patients, relationships, and challenges in the hospital, including emotions and self-care, during these group sessions was restorative for our clinicians [22].

By revealing a more vulnerable and honest version of ourselves, the colleagues around me could feel more able to share the difficulties of working in healthcare that up until now, they had chosen to conceal. My experience is that clinicians want to disclose to, and receive support from, colleagues rather than mental health professionals [18]. The groups were accessed by a range of clinicians across the traditional medical hierarchy (interns to senior consultants, heads of divisions and clinical units, up to professorial level), and were well attended [8]. The sessions also were often extended beyond the appointed end, as people stayed, milling around still wanting to talk. I regularly received positive feedback from session participants on how helpful it was to just talk about what was happening for them. However, it is important to acknowledge that the stressors we were under during the pandemic in a limited resource setting made protecting everyone from all negative mental health outcomes impossible, despite our best efforts. There are circumstances in healthcare that will overwhelm all, despite resilient clinicians, and good psychological support.

Emotional stressors in the medical field are occupational hazards, not mental health problems [5]. Programs relying on individualized mental health interventions alone without including peer support are frequently not used by the clinicians who need them [5]. Clinicians are more likely to accept support from colleagues who understand their specific environmental stressors. The collegial support team-building model also reduces the stigma associated with receiving support [18]. Seeing that colleagues understand your emotional responses and that they have had similar experiences reduces the feelings of isolation and shame associated with feeling distress [5]. The peer support sessions did and have not replaced individualized mental healthcare when needed or requested. In fact, the support sessions and easy access to myself, the psychiatrist within the medical teams facilitated onwards referral to other mental health professionals. Working alongside a psychiatrist in the medical team and wards has played a role in normalizing mental healthcare. This intervention also draws attention to the importance of integrating liaison psychiatry within clinical teams, and during future emergencies and health system shocks, thereby facilitating trust and a space for providing emotional support to patients and colleagues founded in shared experience [18]. Arguably the closest type of intervention to Balint groups (rooted in unidisciplinary (physicians only), with closed membership), are Schwartz Rounds (organization-wide ‘all-staff’ open membership forums to share stories about the emotional impact of providing patient care). In particular, both are ongoing group programmes in which challenging/rewarding experiences about delivering patient care are shared and discussions are facilitated, and both provide the opportunity to give and/or receive peer support in safe and confidential environments. However, Schwartz Rounds offer an organization-wide opportunity for all staff to attend, and attendees can choose to be silent listeners. The addition of Schwartz Rounds may further help ensure optimal outcomes.

Given our better understanding of what worked during the pandemic, and the increasing body of knowledge of the psychological experience of working during COVID-19, we would do well to take the lessons we learned forward into a new way of leading clinicians.

### Benefits of a changed culture: improved well-being, mental health, and reduced risk of burnout

I believe that social connections, friendships, and a feeling of belonging in the workplace is protective of our mental health. Two key elements are required for social connections to happen organically and for them to be maintained. First is continuous unplanned interaction and second, shared vulnerability. Our hospital management team contributed to raising the awareness and importance of the mental health of clinicians with a mental health awareness campaign, which included helpful information about where and how to seek individual counselling. In the hospital, we are in a lot of environments that allow for continuous unplanned interaction; however, these corridor conversations, for example, are not sufficient to create a sense of connection and belonging. In addition, we created protected time for teams to have the opportunity to share vulnerability. Clinical teams need to engage in the active process of ‘teaming’, which occurs when diverse clinicians are brought together for regular face-to-face contact, which stabilizes team membership and facilitates psychological safety. ‘Teaming’ allows clinical units to adapt better to chaotic environments, and to the demands of increasingly complex patient care [6].

Furthermore, additional benefits to a psychologically safe environment within the healthcare institutions include an improvement in well-being, and a reduction in burnout [23].

Vulnerability is an important component of ‘teaming’, and the emotion we experience during times of uncertainty, risk, and emotional exposure [24]. Translated to the clinical context, vulnerability is the doctor’s perception and exposure of emotions essential to connecting with colleagues, understanding patients and their problems, and to recognize the distinctive relational character of practicing medicine. Importantly, vulnerability is not oversharing; it does not require us to reveal all the difficulties in our personal lives to our colleagues. Vulnerability is being okay with showing colleagues that you don’t know everything. It is modelling striving to do what is right in the clinical setting over being right. It is having the courage to have tough conversations and to ask difficult questions knowing that you are going to have to hold the answers in that space. It is showing tenderness and choosing humanness over the archetypal doctor stereotype. It is asking a colleague if they are okay when you can see that they are not. It is having the courage to listen and not to give advice and being a steward of our colleague’s stories. It is getting to know and see and care for the people that you work with. It is showing up and being seen, bringing your real self, not the self moulded by generations of medical hierarchy and culture. It is about letting our colleagues who are in a position of perceived less power than ourselves see our emotions, see our tenderness, see our worry, see our ‘not knowing’, but then see how we manage those feelings and those difficult situations where we aren’t sure what to do. It is embracing being more fully attuned to the patient whose suffering and isolation demands that we open the heart of our professional being [17]. Being vulnerable with each other in the hospital setting is an absolute requirement for real connection with our colleagues.

Connection is in our neurobiology and why our experiences of disconnection can lead to feelings of loneliness, distress, and powerlessness [24]. Connection is a schism, being a risk that we can engage in, but also the most caring and important behaviour that we can engage in. It is both frightening and wonderful at the same time. What we want is to feel a part of the hospital in which we work, to experience real connections with colleagues. Connections to our colleagues gives us more freedom to express our individuality without fear of jeopardising our sense of belonging. Belonging is a practice that requires us to be vulnerable and connected, to get uncomfortable and to learn how to be present with colleagues without sacrificing who we are in the context of the medical culture within which we work. Belonging is a feeling of psychological safety, of being accepted, included, respected, and contributing to your clinical team. Psychological safety is associated with improved team learning and performance, making psychological safety particularly important within healthcare organizations to provide safe care for patients [6].

## Conclusion

What sustained me, and what I will hold on to as a result of this extraordinary time, are the colleagues who survived this with me. We looked out for each other and faced this catastrophe together. The connection and sense of belonging within the team provided the strongest protection against despair and the strongest antidote to the frontline experience [18].

I believe my work with my colleagues has been helpful, and the feedback I have received has been positive, but the experience has given me a window into longer-term questions regarding the well-being of clinicians in an under-resourced healthcare system in a violent and unequal country. My colleagues and I deal with and embody the consequences of a society which is not healthy. It is well established that clinicians migrate from South Africa and similar countries to wealthier countries for a range of reasons, including the stressed health system itself.

Going forward, each of us, regardless of seniority, needs to take responsibility for a change in medical culture. We as clinicians must create time for attending weekly peer support sessions, where we can, as clinical teams, explore parts of ourselves together, think and connect together within our unit/division/department. We need to create weekly spaces that feel nurturing and safe, that allow us to reveal parts of ourselves, to be vulnerable with each other in a way that encourages and maintains psychological safety, social connections and a sense of belonging. So ask yourself, where do you belong? Do you feel a sense of belonging in your department, your division or your research unit? Ask yourself, who are *you* nurturing? But not only that, who is nurturing *you*? Each of us must take responsibility to create these spaces to facilitate this bio-directional nurturing and sharing of vulnerabilities, sharing of parts of each other, which drives a sense of belonging.

## Data Availability

No data set or material involved.
